# Minocycline Impact on Redox Homeostasis of Normal Human Melanocytes HEMn-LP Exposed to UVA Radiation and Hydrogen Peroxide

**DOI:** 10.3390/ijms22041642

**Published:** 2021-02-06

**Authors:** Jakub Rok, Zuzanna Rzepka, Mateusz Maszczyk, Artur Beberok, Dorota Wrześniok

**Affiliations:** Department of Pharmaceutical Chemistry, Faculty of Pharmaceutical Sciences in Sosnowiec, Medical University of Silesia, 41-200 Sosnowiec, Poland; zrzepka@sum.edu.pl (Z.R.); d200888@365.sum.edu.pl (M.M.); abeberok@sum.edu.pl (A.B.); dwrzesniok@sum.edu.pl (D.W.)

**Keywords:** minocycline, antioxidant, oxidative stress, melanocytes, UVA radiation, antioxidant enzymes

## Abstract

Minocycline is a semisynthetic tetracycline antibiotic. In addition to its antibacterial activity, minocycline shows many non-antibiotic, beneficial effects, including antioxidative action. The property is responsible, e.g., for anti-inflammatory, neuroprotective, and cardioprotective effects of the drug. However, long-term pharmacotherapy with minocycline may lead to hyperpigmentation of the skin. The reasons for the pigmentation disorders include the deposition of the drug and its metabolites in melanin-containing cells and the stimulation of melanogenesis. The adverse drug reaction raises a question about the influence of the drug on melanocyte homeostasis. The study aimed to assess the effect of minocycline on redox balance in human normal melanocytes HEMn-LP exposed to hydrogen peroxide and UVA radiation. The obtained results indicate that minocycline induced oxidative stress in epidermal human melanocytes. The drug inhibited cell proliferation, decreased the level of reduced thiols, and stimulated the activity of superoxide dismutase (SOD), catalase (CAT), and glutathione peroxidase (GPx). The described changes were accompanied by an increase in the intracellular level of ROS. On the other hand, pretreatment with minocycline at the same concentrations increased cell viability and significantly attenuated the oxidative stress in melanocytes exposed to hydrogen peroxide and UVA radiation. Moreover, the molecular docking analysis revealed that the different influence of minocycline and other tetracyclines on CAT activity can be related to the location of the binding site.

## 1. Introduction

Oxidative stress is defined as an imbalance between the generation of reactive oxygen species (ROS) and cellular mechanisms of antioxidant defense [1]. Reactive oxygen species involve among others: superoxide radical anion, hydrogen peroxide, and hydroxyl radical. ROS are transformed by antioxidant enzymes, i.e., superoxide dismutase (SOD), catalase (CAT), and glutathione peroxidase (GPx) into less reactive and less harmful molecules [2,3]. ROS play different and important roles in human physiology, pathology, and pharmacology, e.g., in cellular signaling, lipid metabolism, cell proliferation, differentiation, migration, and apoptosis [4,5,6]. However, their reactivity may cause the oxidative damage of most biomolecules, including nucleic acids, proteins, lipids, amino acids, or carbohydrates, leading to the cytotoxic effect [7]. Overproduction of ROS is observed in many chronic and acute disorders, including cardiovascular, neurological, and dermatological diseases [8,9,10,11,12,13,14]. Moreover, oxidative stress is often responsible for some of the adverse drug reactions, including phototoxicity [15].

Tetracyclines belong to drugs that have been arousing the interest of scientists in recent years. Their good toleration, safety, broad spectrum of action, high efficacy, and low cost of therapy have contributed to frequent and widespread use of the antibiotics in medicine and veterinary. Infections treated with tetracyclines involve, among others, anthrax, chlamydial infections, community-acquired pneumonia, Lyme disease, cholera, syphilis, plague, and periodontal and dermatological infections [16]. In addition to the antibacterial activity, tetracyclines show many non-antibiotic effects, which allow tetracyclines to be used in various dermatologic (e.g., acne, rosacea, sarcoidosis, miscellaneous dermatoses, Kaposi’s sarcoma) and non-dermatologic diseases (e.g., rheumatoid arthritis, scleroderma, cancer, periodontitis) [17,18,19].

Minocycline (7-dimethylamino-6-dimethyl-6-deoxytetracycline), due to its semi-synthetic origin, belongs to the second generation of tetracyclines. The drug is characterized structurally by the presence of two dimethylamine substituents: at 4th and 7th positions. Minocycline has highly profitable pharmacokinetic properties. It is very well absorbed (95–100%), mainly in the stomach, duodenum, and jejunum. Its lipophilicity and the volume of distribution (80–115 L) indicate excellent tissue penetration and passing through the blood-brain barrier [20]. These features in combination with the broad-spectrum of non-antibiotic activities make minocycline a molecule with great therapeutic potential. Minocycline is usually used as oral dosage forms, such as capsules or tablets, and intravenous injections. However, minocycline 4% foam, a new topical form, was approved recently to minimize adverse reactions associated with the oral application of the drug. The foam was introduced for the topical treatment of inflammatory lesions of nonnodular moderate to severe acne vulgaris in adults and pediatric patients 9 years of age and older [21].

One of the distinctive features of minocycline is the antioxidant activity. It is believed that the property is partially related to the chemical structure of the drug, especially with the multi-substituted phenol ring as well as the unique 7-dimethylamino group. The mechanism of the antioxidant action of minocycline involves among others direct ROS scavenging (e.g., superoxide anion and hydrogen peroxide) and iron chelation [22,23]. The action is considered to be partially responsible for the anti-inflammatory, neuroprotective, and cardioprotective effects of the drug [24,25,26,27].

The use of minocycline, as in the case of other medicinal substances, may lead to side effects. During long-term pharmacotherapy with minocycline, pigmentation disorders of the skin are observed, manifested by blue, brown, or black discoloration. Pathogenesis of minocycline-induced pigmentation is diverse and includes: (i) deposition of insoluble complexes of minocycline with iron, (ii) deposition of pigmented and reactive metabolites of the drug, and (iii) the elevation of melanin level [28,29,30]. Increased synthesis of melanin—biopolymer produced by highly specialized skin cells—melanocytes, is usually a defensive reaction to exposure to harmful factors, like UV radiation or reactive oxygen species [31]. This protection mechanism is a result of various and unique properties of melanin. Melanin biopolymers act as physiological redox buffer, absorb UV radiation, scavenge for free radicals, chelate metal ions, and prevent enzymatic lysis and damage by oxidants [32,33]. However, melanin can also form complexes with drugs, including tetracyclines, and contribute to their accumulation in pigmented tissues, which in turn may be related to a higher risk of side effects [34,35].

Considering the above problems, a question about the influence of minocycline on the ROS level in pigmented cells arises. Antioxidant activity of minocycline in reference to observed skin adverse drug reactions creates a need to confirm the effectiveness and safety of minocycline in the treatment of dermatological diseases related to oxidative stress. So far, no studies on the effect of minocycline on redox homeostasis in melanin-producing cells have been performed. Therefore, the study aimed to assess the antioxidant properties of minocycline using the culture of human normal melanocytes HEMn-LP.

## 2. Results

### 2.1. Minocycline Inhibits Proliferation of Human Normal Melanocytes

**Figure 1:**
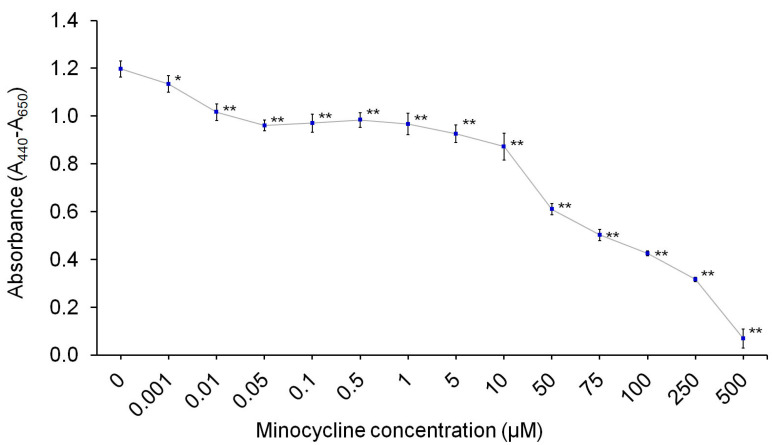
Minocycline inhibits proliferation of human normal melanocytes HEMn-LP cells after 24-h treatment. * *p* < 0.05 ** *p* < 0.005.

**Figure 2:**
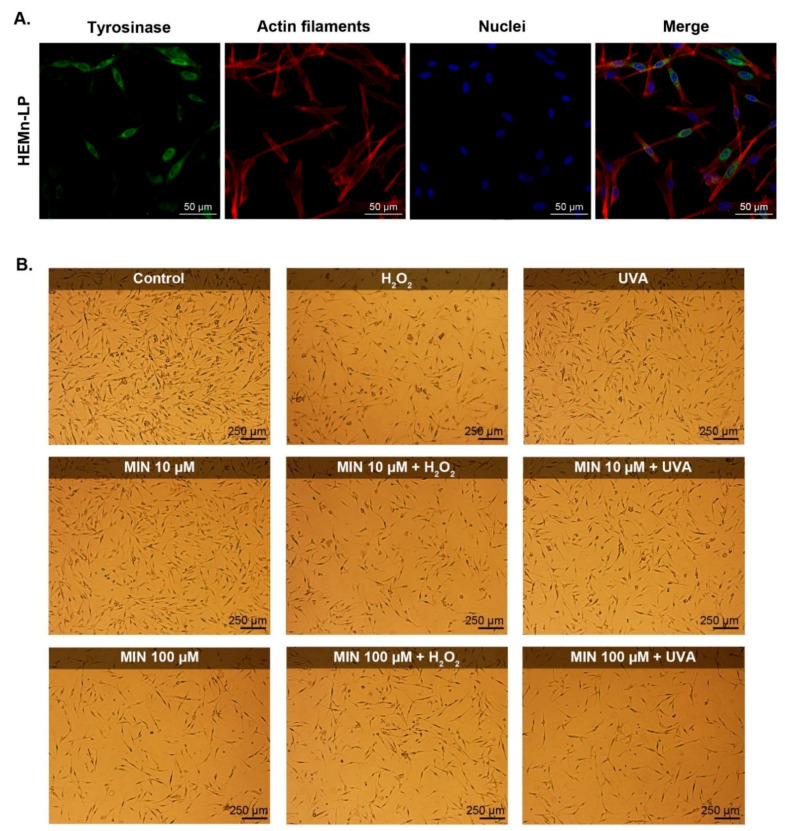
Microscopic evaluation of HEMn-LP cell line. Confocal microscopy imaging of the cells: tyrosinase, actin filaments, and nuclei were stained with anti-tyrosinase primary antibody and Alexa Fluor 488-conjugated secondary antibody, Phalloidin–Atto 565, and DAPI, respectively. Photographs are presented as separate channel as well as a merge image. Scale bar 50 μm (**A**). Microscopic photographs of human normal melanocytes, lightly pigmented exposed to minocycline, hydrogen peroxide, and UVA radiation. Scale bar 250 μm (**B**).

The general influence of minocycline on human melanocytes was tested by use of the cell proliferation reagent WST-1 (Figure 1). The study was carried out on human normal epidermal melanocytes, lightly pigmented. The expression and intracellular localization of tyrosinase, the specific marker of melanocytes, were confirmed by confocal microscope imaging (Figure 2A). The drug was tested in concentrations ranging from 0.001 µM to 500 µM. The obtained results showed that minocycline reduced the cell growth after 24 h in all evaluated concentrations. The effect was proportional to the drug concentration. The read values for a concentration of 50 µM were about 50% lower than control. The highest concentration of minocycline caused a decrease of absorbance by 94.2% (±3.3%) when compared to the control. Minocycline concentrations 10 µM and 100 µM were selected for the following experiments. The results of WST-1 for these concentrations were 72.8% (±4.7%) and 35.5% (±0.9%), respectively.

### 2.2. The Evaluation of Epidermal Melanocyte Culture Treated with Minocycline and Exposed to Hydrogen Peroxide or UVA-Irradiated

**Figure 3:**
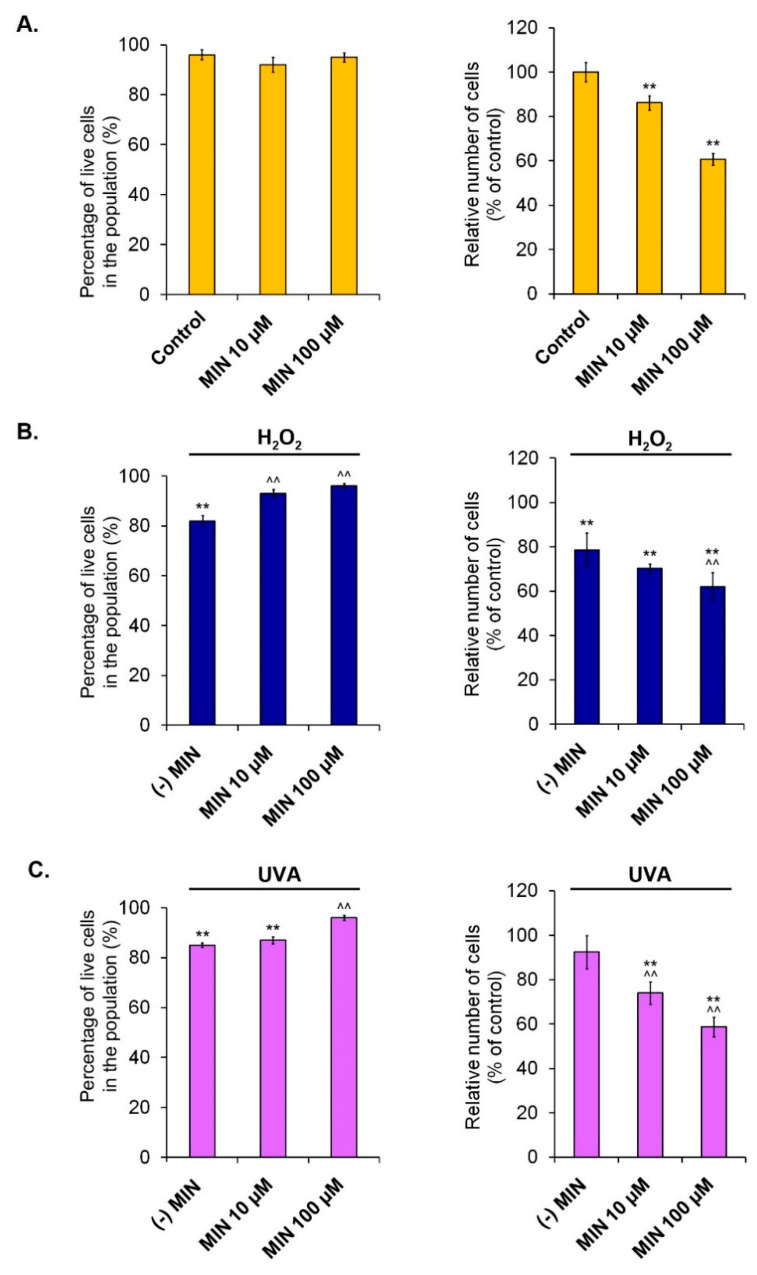
The influence of minocycline on cell number and the viability of: normal human melanocytes (**A**), the melanocytes treated with hydrogen peroxide (**B**), and the melanocytes exposed to UVA radiation (**C**). ** *p* < 0.005. vs. control; ^^ *p* < 0.005. vs. (-) MIN.

The design of all the next experiments involved the influence of minocycline on human epidermal melanocytes as well as pretreatment of the cells with the drug and the following exposure of melanocytes to oxidative stress-inducing factors: hydrogen peroxide or UVA radiation. The evaluation of melanocyte cultures included microscope imaging (Figure 2B), cell counting, and the estimation of the percentage of live cells in the population (Figure 3).

The microscopic images shown indicated that the control culture had the most cells. The control melanocytes had a dendritic appearance and were properly attached and flattened. It was noticed that cells after UVA irradiation, except for a slightly smaller amount, did not differ significantly from the control cells. In turn, the exposure to hydrogen peroxide caused a significant reduction of the cell number as well as induced changes in cell morphology. Most of the cells became thinner and some of them were spherical and swollen. Similar effects were observed in cell cultures treated with minocycline in a concentration of 100 µM. In turn, the tested drug in a concentration of 10 µM did not evoke significant changes in melanocyte morphology and it did not significantly influence the cell morphology exposed to hydrogen peroxide or UVA radiation.

The cytometric evaluation of tested cultures indicated that 10 µM and 100 µM of minocycline reduced cell number in population by 13.9% (±3.3%) and 39.3% (±2.7%), respectively. Simultaneously, it did not decrease the viability of the cells. The percentage of live cells in the population was reduced by hydrogen peroxide (82% ± 2.1%) and UVA radiation (85% ± 0.9%) when compared to the control. It is worth noticing that pretreatment with minocycline in both concentrations prevented the decrease of cell viability in cultures exposed to hydrogen peroxide, and in the higher concentration in UVA-irradiated cultures. In contrast to used UVA radiation, hydrogen peroxide appeared to be the factor that decreased cell number to 78.6% (±7.7%). In the case of cell amount, pretreatment with 100 µM of minocycline additionally reduced the number of melanocytes exposed to hydrogen peroxide to 61.9% (±6.4%). In turn, the number of melanocytes exposed to UVA radiation was decreased by the pretreatment to 73.9% (±5.1%) and 58.7% (±4.4%) for 10 µM and 100 µM of minocycline, respectively.

### 2.3. The Assessment of ROS Level in Melanocytes Treated with Minocycline and Exposed to Hydrogen Peroxide or UVA Radiation

**Figure 4:**
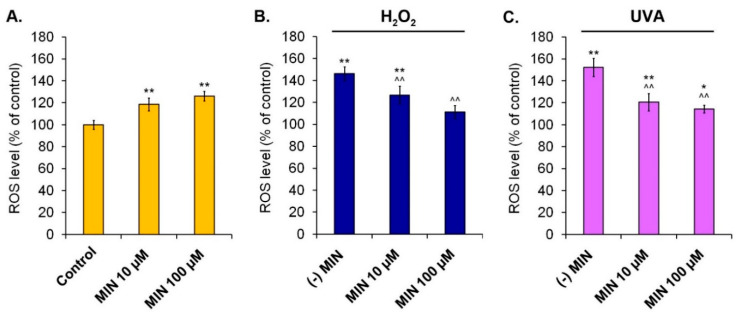
The influence of minocycline on intracellular reactive oxygen species (ROS) level in human normal melanocytes (**A**) as well as melanocytes additionally exposed to hydrogen peroxide (**B**) or irradiated with UVA (**C**). * *p* < 0.05, ** *p* < 0.005 vs. control; ^^ *p* < 0.005. vs. (-) MIN.

The intracellular level of reactive oxygen species was measured using a DCFH_2_-DA reagent. The obtained results (Figure 4) showed that human melanocytes treated with minocycline had elevated levels of reactive oxygen species to 118.7% (±5.9%) and 126.2% (±4.5%) for 10 µM and 100 µM of the drug, respectively. Applied oxidative stress-inducing factors increased the ROS level to 146.1% (±6.4%) for hydrogen peroxide and to 152.4% (±8.2%) for UVA radiation. In turn, the pretreatment of melanocytes with minocycline significantly decreased the amount of ROS, proportionally to the drug concentration. The level of ROS in melanocytes exposed to hydrogen peroxide was 126.8% (±8.2%) and 111.2% (±5.8%) for 10 µM and 100 µM of minocycline, respectively. Analogical results for UVA-irradiated melanocytes were 120.6% (±8.0%) and 114.4% (±3.5%).

### 2.4. The Influence of Minocycline on the Level of Reduced Thiols in Melanocytes Exposed to Hydrogen Peroxide or UVA Radiation

**Figure 5:**
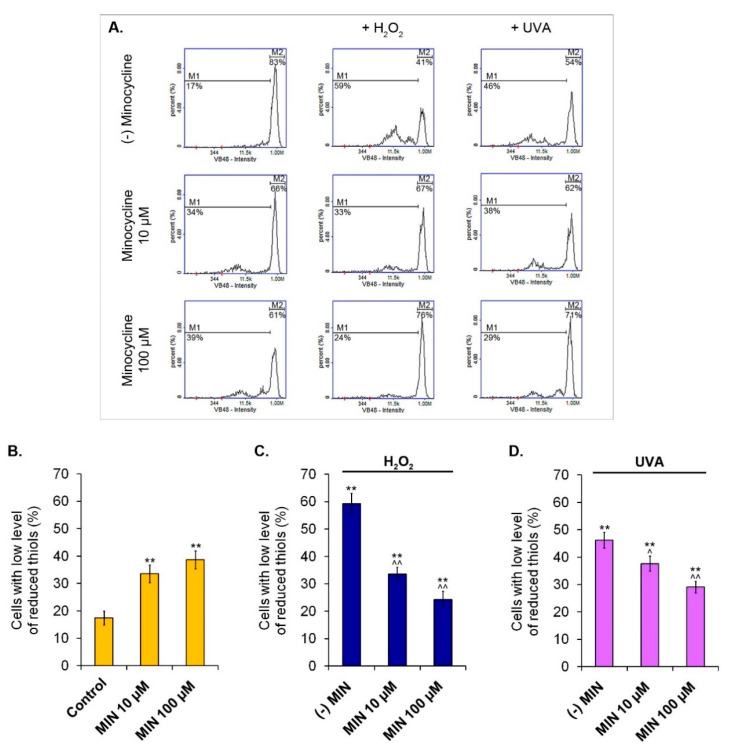
The influence of minocycline on intracellular level of reduced thiols in melanocytes. Representative histograms for individual samples: markers M1 and M2 respond to melanocytes with low and high level of reduced thiols, respectively (**A**). Mean values of the percentage of melanocytes with low level of reduced thiols were presented in the bar graph: for cells treated with minocycline (**B**) as well as additionally exposed to hydrogen peroxide (**C**) and irradiated with UVA (**D**). ** *p* < 0.005. vs. control; ^ *p* < 0.05, ^^ *p* < 0.005. vs. (-) MIN.

The measurement of the intracellular level of reduced thiols was made cytometrically. The obtained results are presented in Figure 5. The percentage of melanocytes with a low level of reduced thiols was increased after the treatment with minocycline, proportionally to the drug concentration. The low level of reduced thiol was stated in 17.4% (±2.6%) of cells in the control, as well as in 33.6% (±3.2%) and 38.7% (±3.3%) of melanocytes incubated with 10 µM and 100 µM of minocycline, respectively. Analogical results for cells exposed only to hydrogen peroxide or UVA radiation were 59.3% (±3.8%) and 46.2% (±2.9%), respectively. The pretreatment with minocycline significantly elevated the number of melanocytes with a high level of reduced thiols. The percentage of hydrogen peroxide-exposed cells with a low level of reduced thiols was 33.6% (±2.4%) and 24.3% (±3.0%) for 10 µM and 100 µM of minocycline, respectively. In turn, the percentage in cell cultures irradiated with UVA was reduced to 37.6% (±2.8%) and 29.1% (±2.1%) for 10 µM and 100 µM of the drug, respectively.

### 2.5. The Activity of Antioxidant Enzymes in Melanocytes Treated with Minocycline and Exposed to Hydrogen Peroxide or UVA-Irradiated

**Figure 6:**
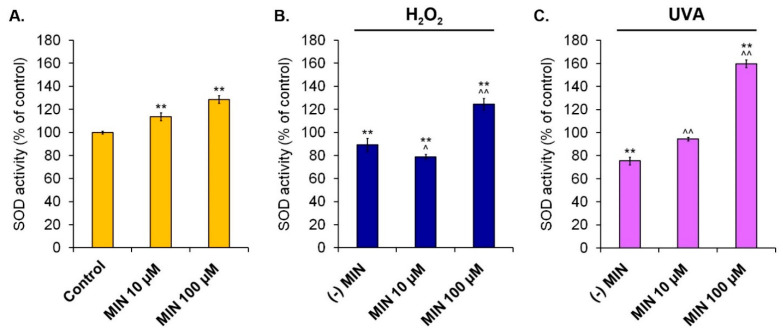
Changes of superoxide dismutase activity in human normal melanocytes treated with minocycline (**A**) as well as additionally exposed to hydrogen peroxide (**B**) or irradiated with UVA (**C**). ** *p* < 0.005. vs. control; ^ *p* < 0.05, ^^ *p* < 0.005. vs. (-) MIN.

**Figure 7:**
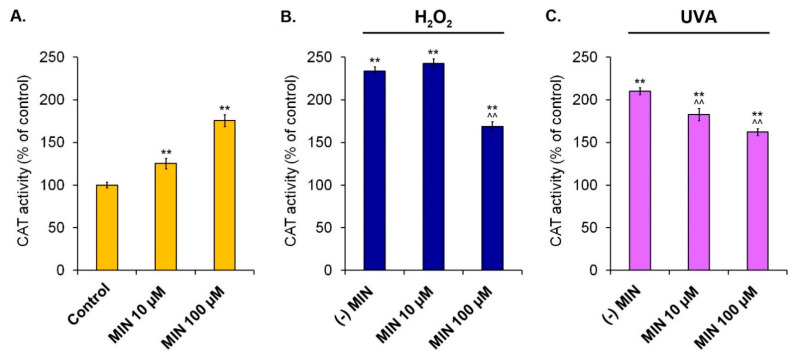
Changes of catalase activity in human normal melanocytes treated with minocycline (**A**) as well as additionally exposed to hydrogen peroxide (**B**) or irradiated with UVA (**C**). ** *p* < 0.005. vs. control; ^^ *p* < 0.005. vs. (-) MIN.

**Figure 8:**
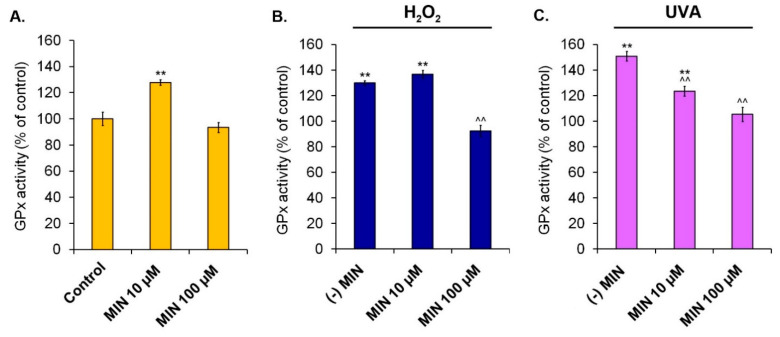
Changes of glutathione peroxidase activity in human normal melanocytes treated with minocycline (**A**) as well as additionally exposed to hydrogen peroxide (**B**) or irradiated with UVA (**C**). ** *p* < 0.005. vs. control; ^^ *p* < 0.005. vs. (-) MIN.

The activity of antioxidant enzymes was measured colorimetrically. Figure 6, Figure 7 and Figure 8 present results for superoxide dismutase, catalase, and glutathione peroxidase, respectively.

The study concerning SOD showed that minocycline augmented the activity of the enzyme, proportionally to the drug concentration. It was found that SOD activity increased to 113.7% (±3.4%) and 128.6% (±3.2%) for 10 µM and 100 µM of minocycline, respectively. In turn, hydrogen peroxide caused the decrease of the enzyme activity to 89.2% (±5.7%). In the case of melanocytes exposed to hydrogen peroxide, the pretreatment with 10 µM of minocycline additionally reduced SOD activity to 78.9% (±2.2%). On the other hand, preincubation of the cells with 100 µM of the tested drug increased SOD activity to 124.6% (±4.9%). The increase of SOD activity after minocycline pretreatment was also observed in melanocytes exposed to UVA radiation. The drug in concentrations of 10 µM and 100 µM caused the elevation of SOD activity in UVA-irradiated cells from 75.6% (±3.2%) to 94.4% (±1.5%) and 159.8% (±3.4%), respectively.

Similarly to the case of SOD, minocycline caused the increase of CAT activity, proportionally to the drug concentration. The obtained results were 125.3% (±6.1%) for 10 µM and 175.7% (±7.0%) for 100 µM. The activity of catalase in melanocytes exposed to hydrogen peroxide was 233.6% (±5.0%). The tested drug in a concentration of 10 µM additionally increased this value to 242.5% (±5.8%). On the other hand, 100 µM of minocycline significantly reduced CAT activity in melanocytes exposed to hydrogen peroxide to 169.0% (±5.5%). It was found that both tested concentrations of minocycline decreased CAT activity in UVA-irradiated cells. Pretreatment with 10 µM and 100 µM of the investigated drug caused the reduction of CAT activity from 210.3% (±4.3%) to 182.9% (±7.3%) and 162.3% (±3.4%), respectively.

The assessment of GPx activity indicated that minocycline only in a concentration of 10 µM caused a significant increase to 127.9% (±2.1%). The highest concentration of the drug did not influence the enzyme activity. It was also noticed that GPx activity was elevated by hydrogen peroxide to 129.8% (±1.9%). The tested drug in a concentration of 10 µM additionally increased this value to 136.7% (±3.1%). On the other hand, 100 µM of minocycline reduced GPx activity in cells exposed to hydrogen peroxide to 92.4% (±4.4%). Similar to hydrogen peroxide, UVA radiation caused an increase in GPx activity. The result was in this case 150.7% (±3.8%). Pretreatment with 10 µM and 100 µM of minocycline decreased the enzyme activity in melanocytes to 123.4% (±4.0%) and 105.3% (±5.6%), respectively.

### 2.6. The Comparison of Molecular Docking of Minocycline and Chlortetracycline to Catalase

**Figure 9:**
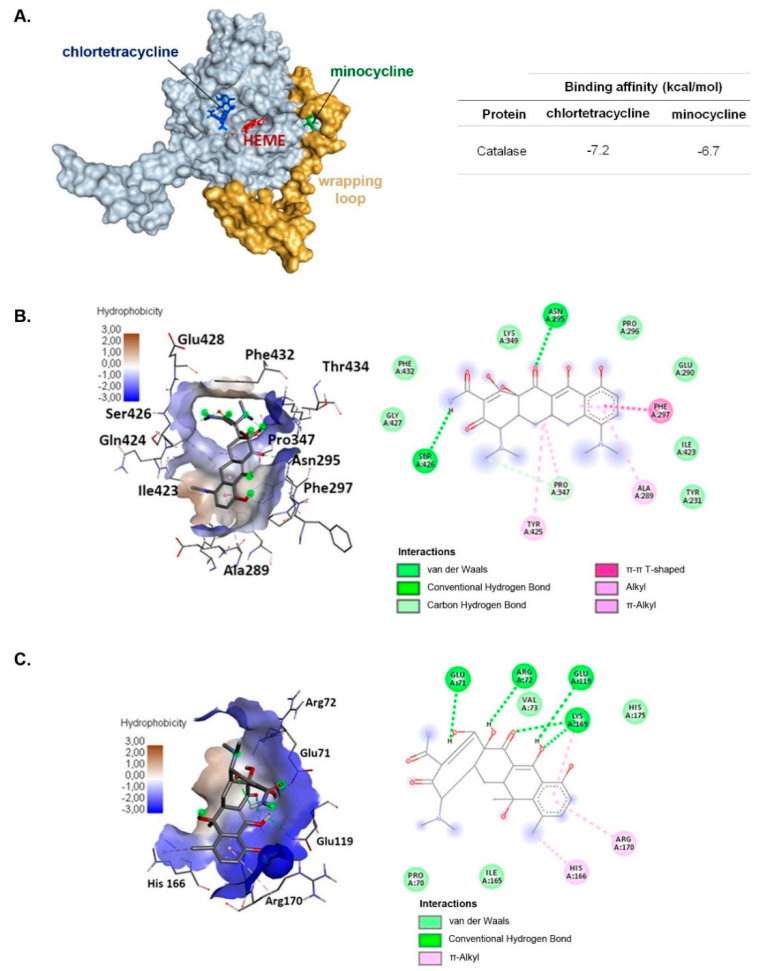
Molecular docking analysis of minocycline and chlortetracycline complexes with human catalase (**A**). The characteristic of interactions between minocycline (**B**) and chlortetracycline (**C**) and the responding binging sites within the enzyme.

The docking study revealed that both minocycline and chlortetracycline had an affinity to catalase. The in silico experiment showed that the primary and best-fit binding site of chlortetracycline was near heme, situated in the catalytic center of the enzyme (Figure 9A). In the case of minocycline, the mean binding site appeared to be located in a groove formed by the wrapping loop of catalase (Figure 9B). Due to the presence of hydrophilic groups in the structures of the ligands, both tested drugs can interact with amino acids residues through hydrogen bonding (6 for chlortetracycline; 3 for minocycline), however, in the case of minocycline hydrophobic bonding is dominant. It is also worth noticing that 7-chloride substituent in chlortetracycline interacts with His166, whereas 7-dimethylamine substituent, characteristic of minocycline, does not participate in any bonding and it can be a steric hindrance in the interaction with the catalytic center. The values of binding energy for minocycline and chlortetracycline in reference to direct interaction with catalase were calculated as −6.7 and −7.2 kcal/mol, respectively.

## 3. Discussion

The skin is the main barrier that protects against numerous environmental physical (e.g., UV radiation) and chemical (xenobiotics) factors. Thus, it is itself exposed to structural and functional damage. Overproduction of ROS in skin cells is one of the reasons for environmental damage of the skin. An increased level of free radicals may lead to the development of many pathologic reactions, including erythema, edema, skin aging, inflammation, allergic reactions, hypersensitivity, keratinization abnormalities, and carcinogenesis [36,37,38]. Moreover, it was found that oxidative stress was related to many cutaneous disorders, such as contact dermatitis, atopic dermatitis, scleroderma, vitiligo, acne vulgaris, psoriasis, and rosacea [10,39,40,41]. Due to this fact, oral and topical antioxidants became an important element of the treatment of dermatological diseases. Molecules with antioxidant properties, e.g., vitamin E, vitamin C, or coenzyme Q10, are known for their ability to neutralize reactive oxygen species, inhibit ROS production, and prevent molecular and cellular oxidative damage [42]. The efficacy of antioxidants in the prevention and therapy of skin disorders prompts the search for new potential drugs reducing oxidative stress.

Antibiotic and non-antibiotic properties of minocycline enable the drug to be used in many dermatological diseases. The antioxidant properties distinguish minocycline from other tetracyclines, including the second generation. A study in rodents showed that the radical scavenging potency of minocycline was 10 times higher than for doxycycline [43]. Moreover, the physicochemical analysis indicated that minocycline appeared to be the most potent free radical scavenger among other tetracyclines, including tetracycline, chlortetracycline, oxytetracycline, and doxycycline [44]. In this study, the scavenging ratio for minocycline (96.1%) was also higher than in the case of well-known antioxidant—ascorbic acid (84.2%). Similar results were presented in the study concerning the neuroprotective action of minocycline [25]. The radical scavenging activity test demonstrated that the potency of minocycline was similar to vitamin E and was higher than in the case of other tetracyclines. It was shown that neuroprotection by minocycline was also related to direct and specific scavenging of peroxynitrite [45]. The antioxidant activity is an important mechanism in the protective action of minocycline among others in sevoflurane-induced hippocampal cell injury, ketamine-induced schizophrenia, cognitive impairments induced by transient cerebral ischemia/reperfusion, Japanese encephalitis virus infection, as well as in diabetes, dyslipidemia, and cardiovascular disease [26,27,46,47,48,49]. Moreover, previously published clinical studies showed a therapeutic potential of minocycline in the treatment of vitiligo, whose pathogenesis involved oxidative stress, depletion of antioxidants, and epidermal cell injury, including melanocytes [50,51]. Minocycline in a dose of 100 mg/day stopped the progression of the disease. There was also skin repigmentation observed in several cases.

Minocycline-induced skin pigmentation is often observed during long-term pharmacotherapy. The phenomenon raises questions about the influence of the drug on melanocyte homeostasis and about the cytoprotective effect of minocycline in pigmented tissues. With regard to the above issues, we decided to investigate the antioxidant properties of the tetracycline on human epidermal melanocytes HEMn-LP.

In the first stage of the study, the impact of minocycline on melanocyte proliferation was assessed in a wide range of concentrations of the tested drug: from 0.001 µM to 500 µM. Based on the obtained results, it was found that minocycline inhibited the proliferation of the tested cells proportionally to the concentration. The EC_50_ value after 24-h incubation was calculated to be approx. 48 µM. It should be noted that the estimated value is significantly lower for the same cell line than previously found for other melanin-binding antibiotics: aminoglycosides (streptomycin—5 mM; kanamycin—6 mM; amikacin—7.5 mM) [52,53,54] and fluoroquinolones (ciprofloxacin, moxifloxacin, and norfloxacin—500 µM; lomefloxacin—750 µM) [55,56,57].

Maximum serum concentration (C_max_) of minocycline depends on the route of administration as well as the dose. It was found that C_max_ after oral administration 200 mg of minocycline ranges from 3.0 mg/L to 3.36 mg/L (from 6.6 µM to 7.3 µM). In turn, intravenous administration of the same dose may result in the increase of C_max_ up to 8.75 mg/L (19.1 µM) [20]. For further studies, minocycline was used at a concentration in the range of plasma C_max_—10 µM and, due to the process of drug accumulation in pigmented tissues, at a concentration of 100 µM. Studies assessing the number of melanocytes and their viability showed that minocycline in the chosen concentration decreased the size of the tested population but did not increase the number of dead cells. The results suggest the antiproliferative effect of the drug on HEMn-LP cells, which was also observed in microscopic images. The effect was proportional to the concentration of the drug and was also observed in melanocytes exposed to hydrogen peroxide and to UVA radiation. Both applied stimulators of oxidative stress decreased cell viability, but a significant reduction of total cell number was observed only in cells incubated with hydrogen peroxide.

Melanocytes, as cells located in the basal layer of the epidermis, are exposed to external damaging and inducing oxidative stress factors. UVA radiation is one of the best-known factors that induce the production of ROS. Moreover, the biosynthesis of melanin in response to, e.g., UV radiation is a sequence of oxidation reactions [58]. The chemical nature of melanogenesis causes the free radicals to be the by-products of the melanin synthesis process. Oxidative stress resulting from the accumulation of ROS in melanocytes may lead to disturbance of the internal homeostasis of these cells, impairing their vital functions and leading to apoptosis and unfavorable transformations [59].

In melanocytes, like in other cells physiologically exposed to oxidative stress, the antioxidant system plays an extremely important role. The system involves small molecules, e.g., glutathione as well as enzymes: superoxide dismutase, catalase, and glutathione peroxidase. Their task is to neutralize ROS like the superoxide radical anion and hydrogen peroxide. It is worth emphasizing that melanin has photoprotective and antioxidant properties. Its ability to absorb and scatter UV radiation makes the biopolymer a barrier limiting the penetration of the radiation and reduces the level of photodamage to the skin [60]. Research indicates that melanin also scavenges and quenches ROS such as superoxide anion [61].

In this study, it was shown that minocycline caused an imbalance of redox homeostasis in HEMn-LP cells. The drug increased intracellular ROS level, the percentage of cells with a low level of reduced thiols as well as changed the activity of the antioxidant enzymes. Moreover, it was found that minocycline, proportionally to the concentration, increased the activity of SOD and CAT in the tested cells. The activity of GPx was raised only in the presence of minocycline in a concentration of 10 µM. The observed changes indicate that the studied drug may contribute to the disturbance of the oxidative-reduction balance and the induction of oxidative stress after 24-h incubation.

Taking into account the inhibited proliferation of cells, it can be assumed that the generation of ROS can be one of the mechanisms of the antiproliferative effect of minocycline on melanocytes HEMn-LP. It is worth noting that the changes have not appeared to be lethal for the cells. Minocycline in a concentration of 100 µM caused a higher decrease in cell number than in a concentration of 10 µM. More potent inhibition of cell proliferation for higher concentration was associated with a higher ROS level and the percentage of cells with reduced thiols. It was stated that both oxidative stress and glutathione influenced cell division. The elevated ROS level may lead to the induction of DNA damage and next to cell cycle arrest [62]. The effect was observed, among others, in human fibroblasts treated with hydrogen peroxide [63]. Growth-arrested cells exhibited predominantly G_1_- and G_2_/M phase distributions. Furthermore, the prolonged mitotic arrest might itself lead to an increase in the cellular levels of ROS [64]. Glutathione is one of the well-known natural antioxidants. It also belongs to essential factors regulating the survival of eukaryotic cells. Most GSH co-localizes in cell nuclei where may regulate cell proliferation. Previously published studies showed that the impairment of cell proliferation could be a result of the depletion of nuclear glutathione [65,66]. Thus, the observed inhibition of melanocyte proliferation may be an effect of drug-induced disturbance of redox homeostasis.

It is difficult to unambiguously indicate the reason or mechanism for minocycline-inducing redox imbalance. Among the possible causes can be mentioned: (i) direct influence of minocycline on the antioxidant system or ROS production, (ii) the ability of minocycline to induce melanogenesis which could be also the source of ROS, and (iii) the impairment of mitochondrial function by the drug [67,68]. The conducted studies showed that minocycline caused, e.g., mitochondrial depolarization and permeabilized the inner mitochondrial membrane by forming ion channels [68,69]. It was stated that mitochondria, as a major source of ROS in cells, regulate cell proliferation [70]. Although mitochondrial ROS can stimulate cell division in physiological conditions, disrupted mitochondria are often related to the inhibition of cell proliferation [71]. The phenomenon was observed in many studies on cancer cells, e.g., for berberine, doxycycline, or derivatives of 1-hydroxynaphthalene-2-carboxanilides [72,73,74].

The possibility of induction of oxidative stress by minocycline in human melanocytes seems to be interesting given the many studies indicating its antioxidant properties. Therefore, the obtained results did not allow to make an unequivocal statement about the effect of minocycline, the experimental model was extended with the induction of oxidative stress by the use of H_2_O_2_ solution or UVA radiation. The previously conducted study on immortalized mouse melanocytes B10BR exposed to hydrogen peroxide indicated that short pretreatment with minocycline could prevent a decrease of melanin synthesis and cell apoptosis via inhibition of JNK and p38 MAPK pathways [75].

The results presented in this study revealed that the exposition of human melanocytes to 200 µM of hydrogen peroxide for 24 h as well as to a dose of 2J/cm^2^ of UVA radiation (irradiation time: 46 min and 18 s) led to a significant increase in ROS production and in the percentage of cells with a low level of reduced thiols. Application of the oxidative stress-inducing factors caused the increase of CAT and GPx activity and the decrease of SOD activity. The biochemical effects were also related to the reduction of total and live-cell numbers in the tested population. Preincubation of the cells with minocycline contributed to the prevention of the changes. Generally, melanocytes pretreated with minocycline had a significantly lower intracellular level of ROS and the percentage of cells with a low level of reduced thiols. Moreover, the drug caused in this case a reduction of CAT and GPx activity as well as an increase of SOD activity and the percentage of live cells. In almost all cases, the observed action of minocycline was proportional to the drug concentration. However, it should be noted that 10 µM of minocycline unexpectedly caused gentle intensification of hydrogen peroxide influence on SOD and CAT activity. The reason for this phenomenon may be too low drug concentration to effectively protect melanocytes against the adverse effects of the oxidizing substance. Thus, the efficacy of the antioxidant action of minocycline could be dependent on a concentration of the drug or an oxidant as well as on the kind of oxidative stress-inducing factor.

Antioxidant activity of minocycline was also tested by Mora et al. on the *Drosophila melanogaster* model [76]. The study involved the stimulation of oxidative stress by manganese. Presented results indicated that minocycline itself caused an increase in SOD activity and a decrease in the level of oxidative stress markers, such as hydrogen peroxide. The drug did not influence CAT activity when compared to the control. The study showed that minocycline treatment of adult male *Drosophila melanogaster* with induced oxidative stress significantly reduced SOD and CAT activity as well as the level of hydrogen peroxide. In general, these findings are consistent with the results presented in this study. Furthermore, it suggests that the antioxidant properties of minocycline may be independent of an oxidative stress inducer.

Based on the obtained results and previously conducted studies, it can be stated that the antioxidant activity of minocycline can be linked to the influence on antioxidant enzymes. The presented study indicated that well-known oxidative stress-inducing factors like H_2_O_2_ and UVA radiation caused a decrease of SOD activity in melanocytes, meanwhile, the effect of minocycline was the reverse. Minocycline-induced elevation of SOD activity was also responsible for the inhibition of oxidative stress and neuroprotection in the process of acute cerebral infarct [77].

The opposite action in relation to minocycline showed also the first generation tetracycline: chlortetracycline which decreased CAT activity in human melanocytes [78]. The demonstrated results of molecular docking suggest the difference arises from the location of the binding site in catalase. Chlortetracycline has a high-affinity binding site close to the heme prosthetic group and probably acts as a noncompetitive inhibitor of the enzyme. It was presented that chlortetracycline formed a hydrogen bond with an arginine residue (Arg72), which also made a salt bridge with heme [79]. It was suggested that this residue assisted in the heme burial into the protein structure and affects its redox potential. Therefore, chlortetracycline binding to Arg72 may affect the activity of the enzyme by inducing changes near the active site. In turn, the binding site of minocycline is placed in the wrapping loop and it does not directly affect the active site of CAT. The possible direct influence of minocycline on catalase may take into account an allosteric effect as well as an interaction with the N-terminal polypeptide chain of another catalase subunit [80].

The presented results as well as previously published studies indicate that the biological action and pharmacological properties of antioxidants are ambiguous and complex. The final antioxidant or pro-oxidant effects may be related to many conditions, such as a dosage of the tested compound, an experimental model, and its redox state. The issue was raised in several studies of resveratrol—one of the most widely and frequently tested antioxidants. Similar to minocycline, the compound is known for its beneficial properties, including antioxidant, anti-inflammatory, and cardioprotective effects. However, under certain conditions, resveratrol increases ROS level and triggers pro-oxidant and cytotoxic effects [81]. The study on the heart, liver, and kidney of rats showed that antioxidant properties of resveratrol were observed only in high lipid peroxidation conditions. In turn, low lipid peroxidation conditions caused that resveratrol worked as a pro-oxidant [82]. Moreover, the experiments on human umbilical vein endothelial cells indicated that resveratrol acted as an antioxidant only at low concentrations. The compound at high concentrations caused an increase in ROS level, redox status, and decreased cell number [83,84]. A similar effect was noticed in the case of coumaric acid [85]. It was also observed that other natural antioxidants, such as chlorogenic, ferulic, and coumaric acid, might show anti- and pro-oxidant properties. The effect was dependent on both the used concentration and the intracellular redox state [86]. Diverse redox properties also showed gamma-tocotrienol and alpha-tocopherol. The study on osteoblast treated with hydrogen peroxide indicated that gamma-tocotrienol became a pro-oxidant at high concentrations instead of preventing oxidative-induced damage [87]. In turn, alpha-tocopherol caused a decrease in plasma and LDL oxidizability only under strong oxidative conditions. Mild oxidative conditions changed the effect to pro-oxidant [88]. Thus, both compound dosage and the redox state of the experimental environment may significantly affect the final action of antioxidants. Therefore, the knowledge about these conditions appears of primary importance, especially when precise redox modulation of cells is needed.

## 4. Conclusions

To summarize, the presented results indicate, for the first time, the influence of minocycline on redox homeostasis and induced oxidative stress in melanocytes HEMn-LP is complex. It was presented that the tested drug, proportionally to concentration, inhibited cell proliferation, decreased level of reduced thiols, and stimulated the activity of antioxidant enzymes. The changes were related to the enlarged intracellular level of ROS. On the other hand, pretreatment with minocycline appeared to be beneficial for melanocytes in the model of stimulated oxidative stress. The drug increased cell viability as well as significantly attenuated oxidative stress in cells exposed to hydrogen peroxide and UVA radiation. Analyzing the obtained results and previously conducted studies, it was concluded that the antioxidant properties of minocycline arose from its ability to induce the activity of superoxide dismutase and catalase. The observed effect of minocycline on the enzymes was reversed to the action of the inducers of oxidative stress. Considering the structure of the tested drug as well as the obtained results, it can be stated that the antioxidant properties of minocycline are unique, however, they may depend on other factors and conditions. Nevertheless, this study confirmed the efficacy of minocycline to prevent the oxidative stress caused by hydrogen peroxide and UVA radiation in human melanocytes.

## 5. Materials and Methods

### 5.1. Chemicals and Reagents

Minocycline hydrochloride, C_23_H_27_N_3_O_7_ x HCl, SIGMAFAST Protease Inhibitor Cocktail Tablets, EDTA-Free and Phosphatase Inhibitor Cocktail 3, DCFH_2_-DA (2′,7′-dichlorofluorescein diacetate), penicillin, amphotericin B solution (250 µg/mL), and phosphate-buffered saline (PBS) were obtained from Sigma-Aldrich Inc. (Taufkirchen, Germany). An M-254 growth medium and a human melanocyte growth supplement-2 (HMGS-2) were purchased from Cascade Biologics (Portland, OR, USA). Neomycin sulfate was acquired from Amara (Kraków, Poland). Trypsin/EDTA was obtained from Cytogen (Zgierz, Poland). Cell Proliferation Reagent WST-1 was acquired from Roche GmbH (Mannheim, Germany). Dulbecco’s phosphate-buffered saline (DPBS), primary anti-tyrosinase monoclonal antibody, secondary antibody conjugated with Alexa Fluor 488 as well as DAPI (4′6-diamidino-2-phenylindole, dihydrochloride) 1 mg/mL in water were purchased from Thermo Fisher Scientific Inc. (Waltham, MA, USA). Solution 5 (400 μg/mL VitaBright-48™, 500 μg/mL propidium iodide, 1.2 μg/mL acridine orange in DMSO), as well as Via-1-Cassettes™ (acridine orange and DAPI fluorophores) and NC-Slides™ A8 were obtained from ChemoMetec (Lillerød, Denmark). The remaining reagents and chemicals were produced by POCH SA (Gliwice, Poland).

### 5.2. Melanocyte Culture

All in vitro studies were performed on human epidermal melanocytes, neonatal, lightly pigmented HEMn-LP (Cascade Biologics, Portland, OR, USA) from passages 6 to 10. M-254 growth medium was supplemented with a human melanocyte growth supplement-2 (HMGS-2) as well as antibiotics: penicillin (100 U/mL), neomycin (10 μg/mL), and amphotericin B (0.25 μg/mL). Melanocytes were cultured in a 5% CO_2_ incubator CB 160 (BINDER, Tuttlingen, Germany) at 37 °C. 

### 5.3. WST-1 Assay—The Assessment of Cell Proliferation

Melanocyte proliferation was evaluated by the WST-1 assay. WST-1 is a slightly red tetrazolium salt that can be reduced to dark red formazan dye by mitochondrial dehydrogenases in viable cells. Briefly, melanocytes were seeded in a 96-well microplate in the amount of 5 × 10^3^ cells/well and cultured for 48 h. The growth medium was then replaced with minocycline solutions at concentrations ranging from 0.001 µM to 500 µM. All the drug solutions were prepared by dilution of minocycline aqueous solution (10 mM) in the growth medium. After 21-h-long treatment, WST-1 reagent was added to the tested culture in an amount of 10 µL/well. The measurement was made after 3 h using microplate reader Infinite 200 PRO (TECAN, Männedorf, Switzerland). Absorbance readings were taken at 440 nm and 650 nm as a reference wavelength. Control samples were normalized to 100%, and all tested samples were calculated as the percentage of the control.

### 5.4. Melanocyte Treatment

Melanocytes HEMn-LP were seeded in Petri dishes in an amount of 1 × 10^6^ cells/dish and incubated with the growth medium for 48 h. Then, the medium was replaced with minocycline solutions. After 24-h treatment, some samples were exposed to UVA radiation (BVL-8.LM lamp, Vilber Lourmat, Marne-la-Vallée, France) using the following parameters: λ_max_= 365 nm, 2 J/cm^2^ (720 µW/cm^2^; irradiation time—46 min. and 18 s) and next incubated for 24 h with the growth medium. Unirradiated melanocytes were cultured with hydrogen peroxide solution (200 µM) or with the growth medium for the same time. Afterward, all cells were trypsinized, centrifuged, suspended in the growth medium, and then counted using Via1-Cassettes™ and a fluorescent imaging cytometer NucleoCounter^®^ NC-3000™ (ChemoMetec, Lillerød, Denmark). The measurement of cell amount was performed 3 times for each tested population using the “cell viability and cell count assays” protocol by NC-3000 image cytometer. The method allows counting cells in the range of 5 × 10^4^ cells/mL to 5 × 10^6^ cells/mL.

### 5.5. Microscopic Assessment of Melanocyte Culture

General characterization of melanocytes HEMn-LP was performed by imaging using the laser confocal microscope Nikon Eclipse Ti-E A1R-Si and Nikon NIS Elements AR software (Nikon Instruments, Amsterdam, The Netherlands). The cells were cultured in sterile coverslips placed in Petri dishes for 48 h. After this time, the cells were fixed with paraformaldehyde (4%) and incubated with primary anti-tyrosinase antibody (1:100) overnight at 4 °C. Next, the cells were stained with DAPI (1:500), Phalloidin–Atto 565 (0.6 µM), and Alexa Fluor 488 conjugated with the secondary antibody (1:200). The staining allowed to image nuclei, actin filaments, and tyrosinase, respectively. Finally, the coverslips were mounted onto microscopic glass slides. Moreover, tested melanocytes after the treatment were imaging using a light inverted microscope Nikon TS100F (Nikon Instruments, Amsterdam, The Netherlands).

### 5.6. DAPI Staining—The Assessment of Cell Number

Melanocytes were seeded in a 96-well dark microplate in an amount of 5 × 10^3^ cells/well and cultured for 48 h. After this time, the cells were treated according to the procedure described in paragraph 5.4. In the next step, the cells were fixed with cold 70% ethanol, washed twice with PBS, and incubated with 20 nM solution of DAPI for 10 min. After the staining, melanocytes were washed again with PBS and analyzed. The intensity of fluorescence (λ_ex_= 358 nm, λ_em_= 461 nm) was measured using a microplate reader Infinite 200 Pro (Tecan, Männedorf, Switzerland). The obtained results were expressed as a percentage of control.

### 5.7. DCFH_2_-DA Assay—The Measurement of Intracellular ROS

DCFH_2_-DA reagent (2,7-dichlorodihydrofluorescein diacetate) was used to estimate the intracellular level of reactive oxygen species. The reagent inside the cells was deacetylated by esterases and oxidized by ROS to the fluorescent form—DCF. HEMn-LP cells were seeded in a 96-well dark microplate in an amount of 5 × 10^3^ cells/well and incubated in the growth medium for 48 h. Next, the cells were treated with minocycline for 24 h. After the treatment, some cultures were irradiated with UVA and then incubated for 24 h with the growth medium. Unirradiated cells were exposed to hydrogen peroxide solution (200 µM) or were cultured with the growth medium for the next 24 h. Finally, 30 min before measurement, the growth medium was replaced with a 20 μM solution of DCFH_2_-DA, and melanocytes were incubated for 30 min. at 37 °C. After the cells had been washed twice with PBS. The intensity of fluorescence (λ_ex_ = 485 nm, λ_em_ = 530 nm) was measured using a microplate reader Infinite 200 Pro (Tecan, Switzerland). The obtained results were normalized to a cell number and expressed as a percentage of control.

### 5.8. Cell Vitality Assay—The Analysis of the Intracellular Level of Thiols

The measurement of the intracellular level of thiols was made using a fluorescence imaging cytometer NucleoCounter^®^ NC-3000™ (ChemoMetec, Denmark). The assay is based on VitaBright-48™—a highly specific dye staining cells with a high level of reduced thiols, such as GSH. Melanocytes after the treatment were suspended in the growth medium in an amount of 2 × 10^6^ cells/mL. Afterward, 10 µL of Solution 5 was added into 190 µL of the cell suspension. Subsequently, the stained melanocytes were loaded into 8-chamber NC-Slides A8™ and were analyzed using the “vitality (VB-48) assay” protocol in the NC-3000 cytometer. The obtained histograms were used to differentiate the subpopulation of cells with high (healthy cells) and low (unhealthy cells) levels of reduced thiols.

### 5.9. Preparation of Melanocyte Lysates

After the treatment procedure, tested melanocytes were suspended in a lysis buffer, containing a phosphatase inhibitor (10 µL/mL) and a protease inhibitor (1.4 mg/mL) dissolved in PBS. The lysates were prepared by freezing cells in liquid nitrogen (−196 °C). Melanocyte lysates were stored at −86 °C until analysis of the activity of antioxidant enzymes.

### 5.10. Pierce™ BCA Protein Assay—The Analysis of Protein Concentration

The concentration of total protein in cell lysates was determined using Pierce™ BCA Protein Assay Kit (Thermo Fisher Scientific Inc., USA), according to the producer instruction. The assay uses the ability of proteins to a reduction of Cu^2+^ to Cu^1+^ in an alkaline medium as well as the selective and sensitive colorimetric detection of Cu^1+^ by bicinchoninic acid (BCA). The measurement of absorbance was performed at 562 nm using a microvolume spectrophotometer DS-11 (DeNovix^®^, Wilmington, DE, USA).

### 5.11. SOD, CAT, and GPx Assay—The Analysis of Antioxidant Enzymes Activity

The activity of antioxidant enzymes, superoxide dismutase, catalase, and glutathione peroxidase, was estimated using commercially available assay kits (Cayman Chemical, Ann Arbor, MI, USA). The assays are based on the colorimetric measurement of the enzyme-catalyzed reaction products. The tests were performed according to the producer instructions and all were described previously [89,90]. All absorbance readings were taken using a microplate reader Infinite 200 Pro (Tecan, Switzerland). The obtained results were normalized to 1 mg of protein.

### 5.12. Molecular Docking Analysis

The three-dimensional (3D) conformers of chlortetracycline and minocycline were obtained from the PubChem database (https://pubchem.ncbi.nlm.nih.gov/) as the SDF files. To prepare ligands before docking, the energy optimization of these models was performed in Open Babel [91], part of PyRx v0.8 virtual screening software [92]. The crystal structure of human catalase was retrieved from Protein Data Bank (https://www.rcsb.org/, PDB ID: 1F4J). Catalase is a homotetrameric protein consisting of four polypeptide chains containing each a heme prosthetic group. For the docking study, three chains (B, C, D) were removed from the 3D model, as well as water molecules. Moreover, polar hydrogens were added to the structure of the protein, which was done in Discovery Studio Visualizer v17.2.0 (Dessault Systems BIOVIA and Discovery Studio Modeling Environment; Release, 2017). AutoDock Vina [93] included in PyRx v0.8 virtual screening software [92] was used in docking studies. To find the most favorable poses for ligands, a docking experiment was conducted within a grid box covering the whole macromolecule with exhaustiveness set at 16. The results of molecular docking studies were visualized in Discovery Studio Visualizer v17.2.0 (Dessault Systems BIOVIA and Discovery Studio Modeling Environment; Release, 2017). The analysis of molecular docking to catalase was performed for minocycline and chlortetracycline.

### 5.13. Statistical Analysis

Statistical analysis of the results was performed using GraphPad Prism 6.01 Software. In all experiments, mean values of at least three separate experiments performed in triplicate (*n* = 9) ± standard deviation of the mean (SD) were calculated. The results were analyzed statistically by one-way ANOVA as well as Dunnett’s comparison test. The Kolmogorov–Smirnov test checked the compliance of the distribution results and the Brown–Forsythe test checked that the variances of the compared groups met the homogeneity assumption. In all cases, the statistical significance was found for the *p*-value to be lower than 0.05.

## Data Availability

The data presented in this study are available on request from the corresponding author.
